# Raising the Digital Profile of Facial Palsy: National Surveys of Patients’ and Clinicians’ Experiences of Changing UK Treatment Pathways and Views on the Future Role of Digital Technology

**DOI:** 10.2196/20406

**Published:** 2020-10-05

**Authors:** Ala Szczepura, Nikki Holliday, Catriona Neville, Karen Johnson, Amir Jahan Khan Khan, Samuel W Oxford, Charles Nduka

**Affiliations:** 1 Faculty Health & Life Sciences Centre for Intelligent Healthcare Coventry University Coventry United Kingdom; 2 Health & Life Sciences Centre for Intelligent Healthcare Coventry University Coventry United Kingdom; 3 Queen Victoria Hospital NHS Foundation Trust East Grinstead, West Sussex United Kingdom; 4 Facial Palsy UK (Charity) Peterborough United Kingdom; 5 Department of Economics, Institute of Business Administration (IBA) Karachi Pakistan; 6 Exercise & Life Sciences, Faculty Health & Life Sciences Centre for Sport Coventry University Coventry United Kingdom

**Keywords:** Bell palsy, facial nerve paralysis, patient experience, treatment pathway, facial exercise therapy, neuromuscular retraining, treatment adherence, digital technology, outcome measures, telerehabilitation, biosensors, COVID-19

## Abstract

**Background:**

Facial nerve palsy leaves people unable to move muscles on the affected side of their face. Challenges exist in patients accessing facial neuromuscular retraining (NMR), a therapy used to strengthen muscle and improve nerve function. Access to therapy could potentially be improved through the use of digital technology. However, there is limited research available on patients’ and clinicians’ views about the potential benefits of such telerehabilitation based on their lived experiences of treatment pathways.

**Objective:**

This study aims to gather information about facial palsy treatment pathways in the United Kingdom, barriers to accessing NMR, factors influencing patient adherence, measures used to monitor recovery, and the potential value of emerging wearable digital technology.

**Methods:**

Separate surveys of patients with facial palsy and facial therapy specialists were conducted. Questionnaires explored treatment pathways and views on telerehabilitation, were co-designed with users, and followed a similar format to enable cross-referencing of responses. A follow-up survey of national specialists investigated methods used to monitor recovery in greater detail. Analysis of quantitative data was conducted allowing for data distribution. Open-text responses were analyzed using thematic content analysis.

**Results:**

A total of 216 patients with facial palsy and 25 specialist therapists completed the national surveys. Significant variations were observed in individual treatment pathways. Patients reported an average of 3.27 (SD 1.60) different treatments provided by various specialists, but multidisciplinary team reviews were rare. For patients diagnosed most recently, there was evidence of more rapid initial prescribing of corticosteroids (prednisolone) and earlier referral for NMR therapy. Barriers to NMR referral included difficulties accessing funding, shortage of specialist therapists, and limited awareness of NMR among general practitioners. Patients traveled long distances to reach an NMR specialist center; 9% (8/93) of adults reported traveling ≥115 miles. The thematic content analysis demonstrates positive attitudes to the introduction of digital technology, with similar incentives and barriers identified by both patients and clinicians. The follow-up survey of 28 specialists uncovered variations in the measures currently used to monitor recovery and no agreed definitions of a clinically significant change for any of these. The main barriers to NMR adherence identified by patients and therapists could all be addressed by using suitable real-time digital technology.

**Conclusions:**

The study findings provide valuable information on facial palsy treatment pathways and views on the future introduction of digital technology. Possible ways in which emerging sensor-based digital technology can improve rehabilitation and provide more rigorous evidence on effectiveness are described. It is suggested that one legacy of the COVID-19 pandemic will be lower organizational barriers to this introduction of digital technology to assist NMR delivery, especially if cost-effectiveness can be demonstrated.

## Introduction

### Facial Palsy

Bell palsy is an acute unilateral paralysis of the facial nerve, resulting in a patient partially, or completely, losing the ability to voluntarily move facial muscles on the affected side of the face [1]. It is the most common acute disorder affecting a single nerve, and its cause is unknown [2]. Each year, the condition affects 11 to 40 people per 100,000 in the population, most commonly in the age group of 30 to 45 years [3]. The annual incidence of Bell palsy in the United Kingdom is currently 37.7 per 100,000 population [4]. Bell palsy represents only approximately 60% of all facial nerve paralysis (FNP) cases [5]. The total number of FNP cases occurring annually in the United Kingdom is estimated to be at least 22,500, and 1 in 60 individuals will be affected over the course of their lifetime [6,7].

Epidemiological studies indicate that this neurological condition occurs more commonly in those with diabetes, obesity, hypertension, and upper respiratory conditions and people who are immunocompromised or pregnant [2,6,8] or following infection by a virus such as herpes simplex [9]. Data from the United States show a recent rise in incidence, possibly linked to increasing rates of herpes infections [10]. Without intervention, some patients will show an element of recovery within 2 to 3 weeks and complete recovery within 3 to 4 months [2,3]. However, although normal facial function is completely restored in approximately 70% of cases, 30% will have a poor recovery [11,12] with facial disfigurement and sometimes facial pain [3,13], and up to 16% of those affected will have residual involuntary movements known as synkinesis [3]. Research shows that people with these residual deficits experience a long-term reduction in quality of life, psychological distress, depression, and social alienation, often relinquishing a previous public-facing role [3,13-16]. As a result, patients with FNP continue to have relatively low public visibility, unless a high-profile international star reveals their own diagnosis [17].

### Available Treatments

Although various treatments are available, uncertainty exists regarding the effectiveness of many of these. Cochrane systematic reviews have confirmed the effectiveness and cost-effectiveness of corticosteroids (prednisolone) administered within 72 hours of onset of symptoms [18-20]. Beyond this initial treatment, for those with incomplete recovery, there are a number of medical options available. Various surgical procedures, together with botulinum toxin injections can attempt to normalize facial appearance [21-25]. However, a Cochrane review of surgical interventions has reported that there is insufficient evidence to decide whether such procedures are beneficial and has also concluded that further trials are unlikely [26].

Physical rehabilitation therapy can be used as an adjunct to medical treatments. The use of facial neuromuscular retraining (NMR) to strengthen muscle and improve nerve function has been evaluated more than other physical therapies [27-32]. A 2011 Cochrane review has concluded that there is some evidence that NMR can improve facial function (for moderate nerve paralysis and chronic cases) and reduce sequelae in acute cases, although it was recommended that both need to be confirmed in randomized controlled trials [33]. An update of this systematic review is currently underway [34]. A recent review of physical therapy combined with standard drug treatment (SDT) has reported evidence of positive effects on grade and time to recovery compared with SDT alone [35].

### Practice Guidelines and Patient Involvement

In the context of an incomplete evidence base, current international guidelines highlight the need to consider patients’ experiences and preferences [2,36]. Recent clinical practice guidelines from the United States, which conclude that physical therapy can provide potential functional and psychological benefit, add that there is a “large role for shared decision making” [2]. Although Canadian guidelines make no recommendation regarding the use of facial NMR in the acute phase, owing to a lack of good quality trials and risk of bias, its use is suggested for patients who do not have complete facial recovery [36]. In the United Kingdom, clinical guidelines produced by the National Institute for Health and Care Excellence (NICE) recommend rapid initial medication (prednisolone) and referral to a range of hospital-based medical specialists [37]. Guidelines for commissioning neurology services in the United Kingdom also include a recommendation to consider new *transformational technologies* [38].

### Objectives

**Figure 1:**
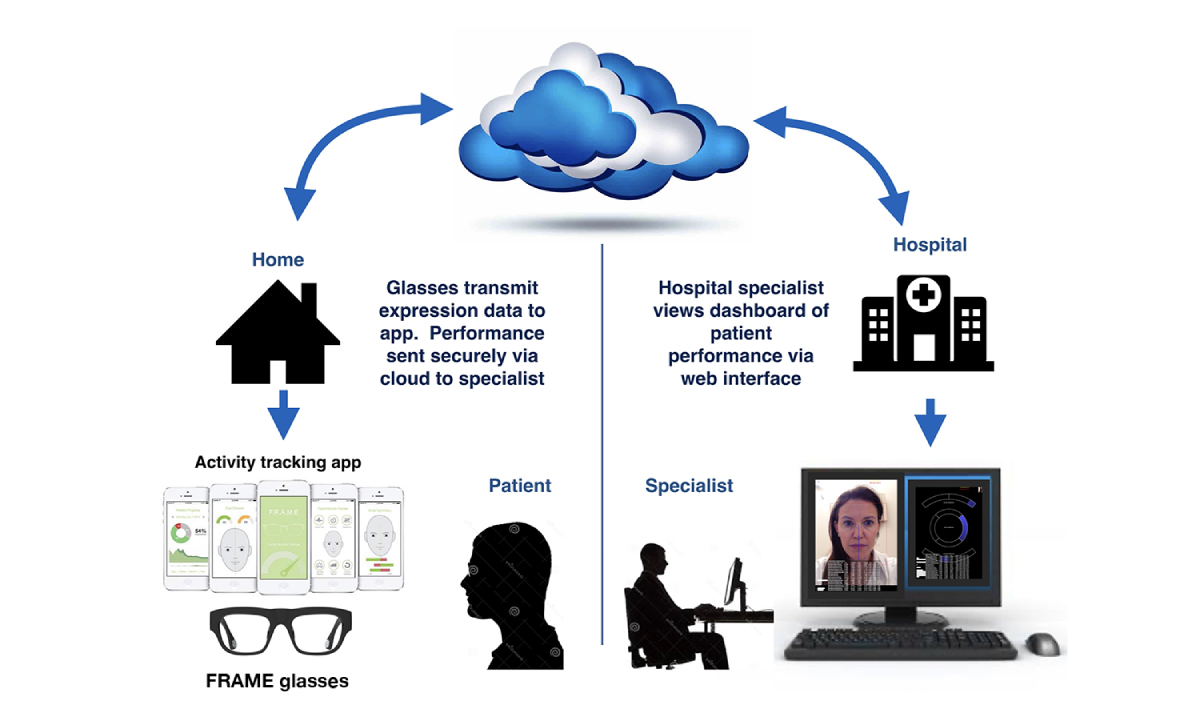
Facial Remote Activity Monitoring Eyewear (FRAME) system overview.

The potential for the “digital patient” to transform the delivery of care has been demonstrated in many clinical areas [39], but there is a lack of evidence for FNP. The aim of this study, conducted in collaboration with patients and specialist clinicians, is to gather evidence about FNP treatment pathways in the United Kingdom, the referral process and timing of NMR, current outcomes used to monitor recovery, and the potential role of digital technology to assist in rehabilitation. The study was conducted as part of a research program (Facial Remote Activity Monitoring Eyewear [FRAME]) funded by the UK National Institute for Health Research (NIHR). The FRAME program aims to develop inconspicuous miniaturized sensor devices in spectacle frames to measure facial movement, providing biofeedback to patients and access for clinicians to outcome data, and to conduct early health technology assessment of the wearable FRAME digital technology shown in Figure 1 [40].

## Methods

### National Surveys

To provide context for technology introduction, national surveys were conducted to explore patients’ and specialist therapists’ experiences of FNP care pathways in the United Kingdom and the potential role of digital technology. Separate questionnaires were co-designed for patients and therapists, piloted, and refined following feedback. Each questionnaire included a mix of closed and open-ended questions (Multimedia Appendix 1) and followed a similar format to enable cross-referencing of some responses [41]. Questionnaires collected demographic details, information on treatment pathways, and ratings of the importance of treatments personally experienced. Respondents were encouraged to add textual comments to expand on responses to closed questions. Further open-ended questions explored the potential value of the emerging FRAME technology. Both questionnaires were uploaded on a web-based platform.

### Recruitment to National Surveys

For patients, an open recruitment strategy was adopted to achieve geographical spread. People with experience of FNP were recruited in collaboration with the national charity, Facial Palsy UK [42]; the patient survey was advertised widely, including via social media. Recruitment of specialist therapists was coordinated through a nation-wide professional group, Facial Therapy Specialists UK [43]; all members were emailed a personal invitation containing a link to the questionnaire, and the initial email was followed by 2 reminders over a period of 5 months.

### Follow-Up Survey

A preliminary analysis of responses uncovered a range of methods used to report treatment outcomes. Therefore, a further survey was conducted to examine these in greater detail, especially any use of validated scales, and whether there is consensus on the definition of a clinically significant change for these measures. A convenience sample of 50 clinicians attending the Facial Therapy Specialists Annual Meeting held in London (October 1, 2018) were invited to complete this questionnaire.

### Analysis of Responses

The closed questions were analyzed using SPSS (version 25, IBM Corporation). Response patterns were summarized using mean and SD or median and IQR, depending on data distribution. Certain variables were grouped to explore changes over time (eg, time since diagnosis). Open-text comments expanding on responses to closed questions and text replying to open questions were analyzed using thematic content analysis [44]. Inductive coding was used, following a flexible analysis approach that helped account for any further themes emerging during the coding process [45]. Data were coded and analyzed for thematic patterns and meanings within the data until saturation was reached.

### Ethics

Ethical approval was granted by the Health and Life Sciences Research Ethics Committee, University of Coventry (ref: P48908).

## Results

### Respondent Rate and Participants

#### National Surveys

The response rate (RR) for patients was calculated based on the number of people accessing the invitation link (after viewing an advertisement) versus the number of patients completing the questionnaire (216/216, 100% RR). Patients were resident in England (all 9 English regions), Scotland, Ireland, and Wales. The RR for clinicians was based on the number of UK therapists contacted who completed the questionnaire (25/49, 51% RR); 5 responses from therapists not currently practicing in the United Kingdom were excluded.

Analysis of patient responses identified that 77.3% (167/216) of patients had acquired FNP as adults, 12.0% (26/216) acquired it at birth or during childhood, 7.9% (17/216) were carers of a child with the condition, and 2.8% (6/216) had another personal or professional connection. Patients with adult-acquired FNP had a mean of 6.96 (SD 7.00) years of experience since first being diagnosed. Table 1 shows that the most common cause of their condition reported by patients was Bell palsy. Specialist therapists were mainly physiotherapists by training (22/25, 88%), with the remainder being speech and language therapists. Clinicians had a mean of 9.72 (SD 7.68) years of experience in treating FNP cases.

**Table 1:** Patients in the United Kingdom with adult-acquired facial nerve paralysis: reported diagnosis and treatment pathways (n=167).

Patient question	Response	Values, n (%)
**Cause of condition (164 responses)**		
	Bell Palsy	89 (54.3)
	Acoustic neuroma or vestibular schwannoma	27 (16.5)
	Ramsay Hunt syndrome	23 (14.0)
	Salivary gland or parotid tumor	4 (2.4)
	Facial nerve neuroma	3 (1.7)
	Birth trauma	1 (0.6)
	Lyme disease	1 (0.6)
	Stroke	1 (0.6)
	Other^a^	8 (4.9)
	Do not know	7 (4.3)
**Treatments provided to date (166 responses)**		
	Advice on eye care	111 (66.9)
	Prednisolone or other corticosteroids	100 (60.2)
	Antivirals	43 (25.9)
	Antibiotics	26 (15.7)
	Botox injections	71 (42.8)
	Facial neuromuscular retraining	101 (60.8)
	Electrical stimulation therapy	35 (21.1)
	Plastic surgery^b^	33 (19.9)
	Psychological therapy (eg, cognitive behavioral therapy)	16 (9.6)
	Other^c^	12 (7.2)
	No treatment^d^	6 (3.6)
**Stage at which first treatment started (164 responses)**		
	Within 72 hours following symptoms	109 (66.4)
	Within 1 month of onset	18 (11.0)
	1-6 months postonset	9 (5.5)
	6-9 months postonset	6 (3.7)
	>9 months postonset	11 (6.7)
	Do not know or other	11 (6.7)

^a^Other causes include the virus of the brain stem, postoperative complications, otitis media, skull fracture, side effect of radiotherapy, and accidental injury.

^b^Plastic surgery includes face lift, brow lift, eyelid surgery, and facial sling.

^c^Other treatments provided include acupuncture, self-funded chiropractic, and massage.

^d^No treatment group includes 2 patients diagnosed with acoustic neuroma or vestibular schwannoma and 4 with Bell palsy (1 assigned to the trial control group).

#### Follow-Up Survey

The follow-up questionnaire was completed by 28 of 50 clinicians (RR 56%); 75% (21/28) were facial therapists, 11% (3/28) were hospital doctors, 8% (2/28) were neurological physiotherapists, and 4% (1/28) were clinical psychologists.

### Treatment Pathways

An analysis of treatment pathways was conducted for patients with adult-acquired FNP. Respondents reported receiving an average of 3.27 (SD 1.60) different treatments following initial diagnosis, most commonly corticosteroids, advice on eye care, and facial NMR, as shown in Table 1.

#### Diagnosis and Initial Treatment

Overall, 66.4% (109/164) of adult-acquired cases who reported the timing of their first treatment said this was within 72 hours of symptom onset (Table 1); for those most recently diagnosed (≤1 year ago), this figure was 91% (31/34) versus 47% (32/68) for patients diagnosed 5 to 18 years ago. The average time to first review of their case was 64 (SD 26.8) days; for those diagnosed ≤1 year ago, this figure was an average of 6 (SD 28.8) days. Fewer than 9.6% (16/166) of patients had been referred for psychological therapy.

#### Referral for NMR

Of 167 respondents with adult-acquired FNP, 98 (58.6%) had been referred for facial NMR, and these patients were treated in 35 different centers. Table 2 shows that nearly half (44/98, 45%) were referred for NMR by a hospital consultant following other treatments; only 28% (27/98) were referred by their general practitioner (GP), and a further 14% (14/98) indicated that they initiated the referral themselves (usually via their GP) following information provided by friends, family, or patient support groups or based on their own research. Therapists reported that their specialist center received a mean of 73.2 (SD 75.5) new facial NMR referrals in an average year, with a large variation between centers (median 30, IQR 123). Table 3 indicates that the mean percentage of referrals from a GP is 37% (SD 32%), with hospital consultants accounting for between 11% (11/98) and 18% (18/98), depending on the specialty.

**Table 2:** Patients with adult-acquired facial nerve paralysis referred for facial neuromuscular retraining (n=98).

Patient question	Response	Values, n (%)
**Referral route to therapy (98 responses)**		
	General practitioner	27 (27.6)
	Self-initiated (usually via a general practitioner)	14 (14.3)
	Plastic surgeon	18 (18.4)
	Ear, nose, and throat specialist	15 (15.3)
	Neurologist	11(11.2)
	Other^a^	11 (11.2)
	Do not know	2 (2.0)
**Any problems with referral** **(97 responses)**		
	Yes	22 (22.7)
	No	69 (71.1)
	Do not know	6 (6.2)
**Feedback provided during therapy^b^(96 responses)**		
	Yes	70 (72.9)
	No	11 (11.5)
	Do not know	15 (15.6)

^a^Referral routes—Other includes solicitor, speech and language therapist, and Botox consultant.

^b^Feedback tended to be given verbally, with the addition of photographic evidence, sharing of electromyography results, scores from the Sunnybrook Scale, or via percent recovered score*.*

**Table 3:** Current referral and treatment pathways reported by facial therapy specialists in the United Kingdom.

Referrals			Values
**Source of referral (24 responses)** **, mean percentage (SD)**			
	General practitioner		37 (32)
	Plastic surgeon		18 (25)
	Ear, nose, and throat specialist		14 (15)
	Neurologist		10 (11)
	Eye specialist		7 (18)
	Other^a^		14 (28)
**Treatments pathway, n (%)**			
	**Wait for first appointment** **following referral** **(weeks; 25 responses)**		
		<1	4 (16)
		1-2	3 (12)
		3-4	8 (32)
		5-6	4 (16)
		8-12	5 (20)
	**Treatments patients receive before referral (25 responses)**		
		Advice on eye care	14 (56)
		Facial neuromuscular retraining	6 (24)
		Prednisolone or other corticosteroids	20 (80)
		Botox injections	12 (48)
		Plastic surgery	11 (44)
		Psychological therapy (eg, cognitive behavioral therapy)	4 (16)
		Other^b^	6 (24)
	**Follow-on referral to other specialists (25 responses)**		
		Ophthalmology	18 (72)
		Botox injections	19 (76)
		Psychological therapy	16 (64)
		Surgery (dynamic facial reanimation)	13 (52)
		Other^c^	7 (28)
	**Feedback provided to referring clinician (25 responses)**		
		Feedback on progress and final outcome	13 (52)
		Feedback on final outcome only	10 (40)
		No feedback provided	2 (8)

^a^Other sources of referral: community pediatricians, neurosurgical or maxillofacial consultants, physiotherapists, speech and language therapists.

^b^Other prior treatments: blood tests, magnetic resonance imaging, electromyography, referral to peer support group, education, soft tissue mobilization, facial massage, and taping.

^c^Other follow-up referrals: radiology; nerve conduction studies; maxillofacial; ear, nose, and throat; speech and language therapy; restorative dentistry; vestibular physiotherapy; and audiology.

**Figure 2:**
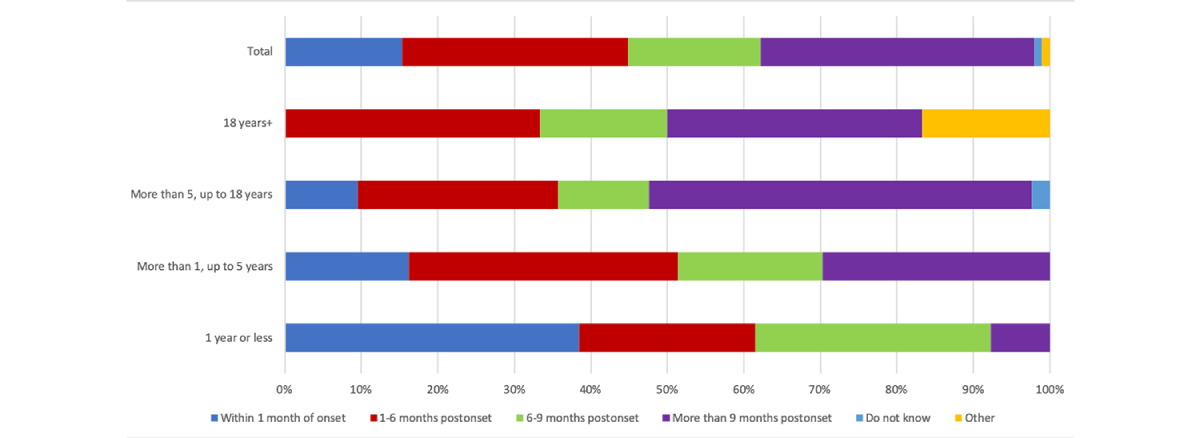
Timing of referral for tailored facial exercise therapy following onset of symptoms.

Figure 2 shows that the referral for NMR occurs at different points following the onset of symptoms. Overall, 15% (15/98) of adult cases were referred within 1 month; 38% (8/21) of people diagnosed most recently (≤1 year ago), compared with 0% for those diagnosed >18 years ago. Once referred, patients reported that they had to wait an average of 7.14 weeks (range 0.5-23 weeks) for a first appointment. Table 3 shows that therapy centers currently record a mean wait time of 4.67 (SD 3.35) weeks. One in 4 adult patients (22/97, 23%) described experiencing difficulties with their referral, most commonly owing to problems accessing National Health Service (NHS) funding, a shortage of specialist therapists, or difficulties in persuading their GP to refer. Overall, 80% (20/25) of centers were aware that some patients experienced difficulties; the 2 main causes identified were limited awareness of facial NMR among GPs and a shortage of NHS funding.

#### Other Treatments

Therapists reported that before referral for NMR, their patients usually received a number of other treatments, as detailed in Table 3, most commonly corticosteroids, advice on eye care, Botox injections, and plastic surgery. In addition, 24% (6/25) of centers reported receiving referrals following failed NMR, presumably provided by a nonspecialist. On completion of facial NMR, 64% (16/25) of centers regularly refer some patients for psychological therapy, 72% (18/25) to ophthalmology, and 52% (13/25) to surgery (dynamic facial reanimation). Although several specialties are involved in the treatment pathway, only 52% (13/25) of therapists had participated in a multidisciplinary team (MDT) review. Similarly, only 23.0% (38/165) of adult patients were aware of such a review of their case, and a further 24.8% (41/165) were uncertain.

#### Views on Treatment

When asked to rate the importance of various treatments, based on their own experience, both patients and therapists rated the same 6 most highly, although median scores indicated little discrimination among them. Patients’ scores produced the following ranking: 1st—advice on eye care, 2nd—facial NMR, 3rd—psychological therapy, 4th—corticosteroids, 5th—Botox injections, and 6th—plastic surgery. Therapists identified a similar order, although they ranked psychological therapy lower as 4th and surgery higher as 5th. All other treatments were rated as important by fewer than 4% of respondents.

### Adherence to NMR

Adult patients reported varying levels of adherence to their prescribed NMR program, with only 33% (32/97) recording very high levels, 41% (40/97) reporting medium-to-high adherence, 21% (20/97) recording poor-to-medium levels, and 5% (5/97) being uncertain about adherence. An analysis of patients’ open-text comments identified 3 main barriers, regardless of the level achieved: “difficulties in fitting facial exercises into daily life,” “using a mirror while completing exercises,” and “insufficient regular follow-up.” Patients who reported a very high level of adherence described 3 further facilitating factors: “observing improved outcomes in self,” “belief that the treatment will work,” and “observing positive outcomes in others.” These patients also highlighted a common fear that acted as a spur: “scared of not recovering.” An analysis of comments made by the medium-to-high adherence patient group uncovered 2 additional demotivating factors: an “inability to see any improvement” and “lack of funds to travel for check-ups.” In terms of travel, distances can be considerable. Although most adult patients (75/97, 81%) were referred to a center within their own region, 9% (8/93) reported traveling ≥115 miles to reach a specialist center. Among patients with poor-to-medium adherence, 2 further barriers were identifiable: “pain associated with facial exercises” and “exercises can be tiring”, possibly indicative of poor execution of a prescribed facial exercise regime.

When asked to describe their patients’ level of compliance, 36% (9/25) reported very high levels, 56% (14/25) reported medium-to-high adherence, 4% (1/25) recorded poor-to-medium levels, and 4% (1/25) were uncertain about adherence. Interestingly, therapists appear to have underestimated the level of poor-to-medium adherence, perhaps suggesting overoptimistic feedback by patients. The thematic content analysis shows that therapists identify the same 3 barriers limiting adherence (ie, “fitting exercises into daily life,” “lack of evidence of improvement,” and “use of a mirror”). Unlike patients, therapists did not identify patients’ travel costs. However, they did additionally think that the timing of other treatments influences adherence, for example, “poorer adherence post-surgery because this may be viewed as the primary treatment for managing their condition” and “Botox given too early may reduce compliance if patients rely on this to relieve feelings of tightness and synkinesis.”

### Feedback on Recovery

Table 2 shows that 73% (70/96) of patients received regular feedback on their recovery during the course of treatment. This is provided in a number of ways with no consistent pattern nationally. Table 3 indicates that the regularity of feedback to referring physicians varies; 52% (12/25) of therapists provide feedback throughout treatment, but 40% (10/25) only report a final outcome. Final discharge summaries also vary, some include validated measures such as the Sunnybrook Scale and others provide photographs showing the patient’s progress.

In response to the follow-up survey, clinicians reported the experience of using various methods for recording treatment outcomes ranging from photographic evidence to recognized disease-specific scales. When asked to name scales they had used, 17 different instruments were identified (Table 4). The most frequently cited was the Sunnybrook Scale (26/28, 93% of respondents) [46], followed by the Facial Disability Index (FDI) identified by 54% (15/28) of respondents [29], and the House-Brackmann (HB) Scale identified by 50% (14/28) of clinicians [47]. Relatively few respondents were able to define a clinically significant change for these measures: 27% (7/26) for Sunnybrook, 7% (1/15) for FDI, and 21% (3/14) for HB. Where answers were provided, there was no consensus.

**Table 4:** Follow-up survey: outcome measures and minimum clinically significant change (n=28).

Outcome measure	Experience of use, n (%)	Minimum clinically significant change
Sunnybrook Scale	26 (93)	Change by 10 points (4 respondents) Change by ≥5 points (1 respondent) 5:1 change (2 respondents) No comment (19 respondents)
Facial Disability Index	15 (54)	Change by ≥5 points (1 respondent) No comment (14 respondents)
House-Brackmann Scale	14 (50)	Change by 1 grade (2 respondents) Grade 3 eye closure or above (1 respondent) Not sensitive enough for therapy outcomes (2 respondents) No comment (9 respondents)
FaCE (Facial Clinimetric Evaluation) Scale	10 (36)	Change by 5 points (1 respondent) Change by 10 points (1 respondent) Change by 15 points (1 respondent) No comment (7 respondents)
Synkinesis Assessment Questionnaire	7 (25)	Change by 1 point (1 respondent) Change by 10 points (1 respondent) Change by 15 points (1 respondent) Other^a^ (1 respondent) No comment (3 respondents)
SF-36 (36-item Short Form Health Survey)	4 (14)	Change by 10 points (1 respondent) No comment (3 respondents)
EuroQol-5D	4 (14)	No comment (4 respondents)
Face-Q^b^; MEEI facegram^c^; CORE-10^d^; MBLF^e^; Lazarini^f^	2 (7)	No comment (2 respondents)
eFACE (electronic Facial Paralysis Assessment)	2 (7)	Change by 10 points (1 respondent) No comment (1 respondent)
Smile Index; CCE angle^g^; Satisfaction with Appearance; Hospital Anxiety and Depression Scale	1 (4)	No comment (1 respondent)

^a^After the treatment score was compared with the opinion of a therapist for training or education purposes.

^b^Developed for facial aesthetic patients, enables users to tailor a version to suit their needs based on over 40 scales measuring a range of concepts important to patients.

^c^FACE-Gram software (MEEI, Boston, Mass).

^d^Comprises 10 items drawn from CORE-OM which is used in evaluation of counselling and psychological therapies in the UK.

^e^French oro-facial myofunctional assessment to quantify impairment and specify motor and functional deficit.

^f^Graphic-visual adaptation of House-Brackmann facial nerve grading for peripheral facial palsy

^g^Angle between the cheilion, contralateral cheilion, and ipsilateral endocanthion.

### Digital Technology

A thematic analysis of open-text comments on the potential value of digital technology identified 4 superordinate themes, as shown in Textbox 1. The first theme, *System*, was predominantly voiced by specialist therapists who highlighted the potential for this technology to help reduce pressure on their time, improve their ability to monitor a patient’s progress, increase coverage, and reduce travel for outpatient reviews. An extra subtheme, expressed by patients within the *System* theme, was that new technology could help raise medical awareness, especially in primary care. A second theme, *Self-management*, was identified by both groups. This focused on improving treatment adherence and emphasized factors such as enabling people to fit facial exercises into their daily routines and providing motivational feedback through regular review of daily performance.

The third theme, *Identity*, emerged mainly from patients’ responses. This highlighted psychological benefits such as improved confidence in people who are frightened to draw attention to themselves and hope in individuals that their condition might improve. The final theme, *Innovation,* encompassed views, such as, that innovation is natural and that it can bring positive societal benefits, together with an awareness that funding constraints limit implementation of innovations. Patients demonstrated a great willingness to support future introduction of digital technology, but they also emphasized the need to raise awareness among GPs alongside implementation. Therapists expressed a similarly positive view of telerehabilitation, with a clear consensus that digital technology could not replace the initial face-to-face consultation because patients need to be carefully trained to be able to complete their exercises properly.

**Themes and subthemes: representative quotes from questionnaires.**

System:

- Therapist time
  “It is a good adjunct to one-to-one therapy for most patients.” (Therapist)
  “I think that for our patient group and geography, Telerehab would work well… we have no dedicated time for facial therapy. It’s included as an acute treatment” (Therapist)
- Monitoring
  “To be able to monitor a patient's progress remotely will be of great value. It also has the potential to generate and collate a lot of objective data on facial function which will be invaluable in efficient data collection for future research.” (Therapist)
  “Any technology in the form of an app and use of EMG biofeedback would be massively useful. This would offer real time feedback about how facial muscles are performing and how successful they are in performing their exercises.” (Therapist)
- Access
  “Our community colleagues I think would benefit greatly and more the patients as many travel from long distances to see the team here as rare specialism.” (Therapist)
  “I am always interested in new advances in medicine. I think if it can provide cover for patients in areas where there is no specialism [in facial neuromuscular], then it will be invaluable.” (Therapist)
  “These are essential for the future of intervention with this patient group especially as there are few specialists and patients are currently having to travel long distances.” (Therapist)
- Medical awareness
  “It [technology] needs to be embraced, further developed and used to educate the medical profession about the condition, its treatments, causes and correct treatment regime.” (Patient)
  “New technology can be useful, but primary diagnosis is important - this [facial palsy] needs more awareness.” (Patient)

Self-management:

- Exercise performance
  “It would benefit some patients and encourage those patients who find it hard to fit exercises into their daily routine.” (Therapist)
  “Using the 'Fitbit' technology whereby users are given rewards for exercising, can check on their daily performance and review weekly summaries of how well they have done. Pop up reminders to do exercises may also [be] very useful.” (Therapist)
- Motivation
  “May be very useful to provide motivational feedback.” (Therapist)
  “Technology could give users rewards for exercising, check on their daily performance and review weekly summaries of how well they have done.” (Therapist)

Identity:

- Confidence
  “Anything that can help the physical issues as well as facial symmetry/making someone feel better about themselves/more confident is so important.” (Patient)
  “If facial palsy patients can be helped by the use of new technologies; they should be. They are hardly likely to fight for treatment as we are traumatised, embarrassed and scared about bringing attention to themselves.” (Patient)
- Hope
  “Personally, I would try whatever technology was available!” (Patient)
  “I am open to trying absolutely anything that could help my symptoms.” (Patient)
  “I would try any new technology in the hope my palsy is improved. Living with it can make you have low self-esteem.” (Patient)

Innovation:

- Natural
  “I think it’s great, it’s good to know there is always research into new ways to aid recovery.” (Patient)
  “I think novel ways of assessing and treating patients using technology must be embraced.” (Therapist)
  “I am all for new technology if it proves helpful to the patient.” (Patient)
- Societal benefit
  “This is exactly what we need! We need to move forward with the times. I feel many new treatment may be expensive initially but if it can change a person life then it is worth it.” (Patient)
  “In the long term it may work out cheaper … [the patient] may have their confidence back and be able to work and contribute back to society.” (Patient)
- Funding
  “This is a very interesting area however costing and funding within NHS would be a concern for clinical use.” (Therapist)
  “I would like to see it made available on the NHS.” (Patient)
  “Financial pressures on health services, the relative rarity of facial therapists, the increasing familiarity / dependence of many people with / on evolving technologies all make it an important part of facial palsy interventions.” (Therapist)

## Discussion

### Principal Findings

This exploration of the experiences of patients with facial palsy and their clinicians provides new information on UK treatment pathways, access to facial neuromuscular retraining, the methods in use to monitor recovery, and patients’ and clinicians’ views on the introduction of an emerging digital technology to support facial NMR. The findings highlight the need to understand patients’ and clinicians’ experiences before introducing such a technology. As funding barriers currently limit access to routine NMR, evidence of cost-effectiveness may be needed before implementation.

#### Treatment Pathways

Although variations were observed in the treatment pathways experienced by patients, there are signs that more recent cases (diagnosed ≤1 year ago) received treatment sooner than cases diagnosed >5 years ago. For the only medical treatment supported by Cochrane systematic reviews (rapid initial prednisolone treatment [19]), we observed high adherence by GPs, especially for patients diagnosed most recently (91%, 31/34 treated). For facial NMR therapy (partially supported by Cochrane review evidence [33]), this study found a move toward earlier NMR referrals in more recent cases. Even so, 1 in 4 patients experienced difficulties with their referral, commonly citing poor GP awareness. The fact that guidelines currently contain no firm guidance on referrals for NMR may offer some explanation [37].

In the United Kingdom, NICE advises that uncomplicated FNP cases can be managed by primary care to include referral to various hospital medical specialists and therapists [37]. This is challenging because an average GP will only see one patient every 2 years [48]. An earlier UK study identified an overall reduction in GP referral rates of patients with FNP to hospital medical specialists over the period 2001 to 2012 [4]. In this study, twice as many referrals to specialist facial therapists were initiated by hospital consultants compared with GP referrals, with patients often prompting the GP referrals. The NICE guidance for GPs does not specifically mention NMR referral, instead indicating a need for confirmatory trial evidence as suggested by the Cochrane review [33]. An update to this review may help inform future guidance on referrals [34]. Finally, although patients in our study ranked psychological therapy third highest in terms of its importance, fewer than 10% had been referred; this is presumably linked to the fact that NICE guidelines only contain a weak recommendation for GPs to consider referral for counseling [37].

Once referred for NMR, access to an appropriate therapist can be problematic. Several patients reported traveling very long distances to reach a specialist center; the cost of travel for regular checkups was also identified as a barrier to adherence by patients. Specialist centers reported receiving patients previously referred to a nonspecialist therapist, reinforcing the need for increased access to appropriately trained professionals [49]. Patient adherence to prescribed facial exercises will influence treatment effectiveness. Both patients and therapists identified the same barriers: fitting exercises into daily life, the use of a mirror, and the need for regular feedback. The fact that clinicians perceive higher levels of adherence than those reported by patients indicates a need for improved monitoring of adherence. Other research has shown that a collaboration between specialists can reduce the burden of long-term disability for acute onset FNP [50]. In this study, although patients experienced care pathways that involved referral to several medical specialties, such collaboration was limited; just over half (52%) of the therapists had any experience of participating in an MDT review, and only 23% (38/165) of adult patients were aware of such a review of their case. Interestingly, reports are now emerging of efforts to integrate physical therapy with treatment by ophthalmologists, oculoplastic surgeons, and ENT and other specialists [51]. It is also considered that MDTs are likely to play an important role in standardizing outcome measures and implementing relevant data collection [52].

Evidence of effectiveness of FNP treatments currently remains reliant on subjective measures, including reduction in *crocodile tears*, *incomplete recovery of motor function*, and *cosmetically disabling sequelae* [19,33]. This study identified inconsistency in the methods used in the United Kingdom to report treatment outcomes. In addition to photographs, various validated scales are used. Among these, the Sunnybrook Scale, mentioned by 96% of specialists, is considered to grade patients in a more objective and continuous manner than the HB Scale, which was mentioned by half [53]. However, FDI, mentioned by 54% of clinicians, better represents impairment, disability, and psychosocial status than the 36-item Short Form Survey (SF-36) health status measure, mentioned by 14% [54]. In addition to variation in the scales used to record treatment outcomes, there was no consensus on what represents a clinically significant change in any scale.

Interestingly, very few clinicians (14%) reported the experience of using EuroQol-5D, the health-related quality of life (HRQoL) measure used for NHS reimbursement decisions [55]. Converted into incremental quality-adjusted life years (QALYs), this is used to quantify long-term treatment outcomes [56]. QALYs are particularly relevant as 30% of patients with FNP will continue to live with reduced HRQoL over the rest of their lives [3,13-15]. In addition, because such individuals may give up their original employment [57], this can lead to a significant long-term societal cost burden, especially if the condition was acquired in early life [58]. An international collaboration has recently been established, focusing on pediatric patients with FNP (using a patient-centered approach, similar to this study) with the aim of comparing FNP treatment pathways, standardizing outcome measurement, and developing value-based reimbursement strategies [59].

#### Digital Technology

To our knowledge, this is the first UK study to explore the potential use of wearable digital technology to support facial NMR therapy. The findings show that patients and therapists both demonstrate a positive attitude toward the introduction of such technology, with patients recognizing benefits centered on better self-management and improved confidence, therapists identifying better monitoring of patients’ progress and reduced work pressures, and both highlighting the potential for improved adherence to facial exercise programs. The main barriers to adherence could all be addressed by an appropriate real time (synchronous) digital solution that addresses patients’ and clinicians’ feedback. A review of telerehabilitation articles has recently concluded that patients’ feedback will help improve future areas of applications, although no FNP telerehabilitation studies were identified [60]. A review of real time, web-based consultation has highlighted a number of general barriers and facilitators [61]. The key barriers and facilitators mirror those found in this study. Asynchronous methods for monitoring FNP treatment outcomes have also recently been evaluated by 2 research teams. Tan et al [62] reported that the assessment of videos using the Sunnybrook and HB scales is as good as a face-to-face assessment, although the lack of real time interaction was judged to limit the value of this approach. Mothes et al [63] found that automatic Sunnybrook grading of photographs using machine learning can deliver fair agreement compared with the subjective rating of the same photographs. Neither study addressed real time monitoring and biofeedback.

### Limitations

A number of limitations should be borne in mind when considering this research. First, patient respondents may not be representative of the wider population because participants were recruited via a specialist support group. Second, there may be recall inaccuracy when participants are asked to provide information sometime after the event. Third, the therapist RR (51% national survey and 56% follow-up survey) means that data may not fully reflect the national picture. Finally, although the greatest care was taken in the questionnaire design, as with all surveys that record individuals’ views, the validity and reliability of the data could not be tested independently.

### Conclusions and Implications for Technology Introduction

To date, little research has explored the potential value of digital technology in assisting facial NMR therapy. This study provides a baseline overview of FNP treatment pathways in the United Kingdom, the factors limiting access to NMR and influencing therapy adherence, the main methods used to record treatment outcome, and the potential role of digital technology. The study indicates that harmonization of outcome measures is required to both strengthen the evidence on treatment effectiveness and to better support MDT management. The main factors limiting NMR adherence could all be addressed through the use of real time digital technology. However, for the type of wearable technology being considered, product design will be an additional factor likely to influence adherence [64], especially in the younger 30- to 45-year age group affected by this condition [3]. However, although the study clearly demonstrates positive attitudes toward the introduction of digital technology, economic barriers may prove to be a challenge. Previous research has identified funding as a barrier for access to surgical treatments for FNP in England [65] and more recently in Wales, Scotland, and Northern Ireland [66]. This study has similarly identified funding barriers to NMR referral. Finally, organizational and cultural factors are acknowledged to act as important barriers to implementation for all digital health innovations [67], often reinforced by policy priorities [68]. One legacy of the COVID-19 pandemic is that health systems worldwide are rapidly adopting digital options in many clinical areas [69]. Thus, barriers to the introduction of digital technology to assist facial NMR therapy may now be lower, especially if cost-effectiveness can be demonstrated.

## Appendix

Multimedia Appendix 1 National Survey Questionnaires.

## Abbreviations

ENT: ear, nose, and throat
FDI: Facial Disability Index
FNP: facial nerve paralysis
FRAME: Facial Remote Activity Monitoring Eyewear
GP: general practitioner
HB: House-Brackmann
HRQoL: health-related quality of life
MDT: multidisciplinary team
NHS: National Health Service
NICE: National Institute for Health and Care Excellence
NIHR: National Institute for Health Research
NMR: neuromuscular retraining
QALY: quality-adjusted life year
RR: response rate
SDT: standard drug treatment
