# Supplementary material for: Raising the Digital Profile of Facial Palsy: National Surveys of Patients’ and Clinicians’ Experiences of Changing UK Treatment Pathways and Views on the Future Role of Digital Technology
Source: J Med Internet Res. 2020 Oct 5;22(10):e20406. doi: 10.2196/20406 (PMC7573702; doi:10.2196/20406)
Supplement: Multimedia Appendix 1 [file jmir_v22i10e20406_app1.docx]

# Multimedia Appendix 1 – **National Survey Questionnaires**

# **1. Patient Questionnaire**

**Facial Palsy – Introduction of Digital Technology**

Facial palsy affects approximately 22,500 people annually in the UK, with the cumulative number of cases since 2000 totalling over one third of a million. Although seventy per cent of cases will achieve complete recovery, it is estimated that the number of people living life with some level of disability during this period is 115,000, including 63,500 with a permanent deficit of facial function

Facial palsy affects both men and women equally, occurring most commonly in people aged between 15 and 60 years, but the condition is commoner in those who are pregnant, have diabetes, or conditions such as hypertension, sarcoidosis and HIV infection. It is estimated that for the 63,500 with a permanent disability, poor recovery leads to the loss of two quality adjusted life years (QALYs) per person.

**Current treatment options for facial palsy**

Currently, oral corticosteroids (Prednisolone) within 72 hours of onset of symptoms are the only treatment recommended by NICE, with strong evidence that this can improve outcome and shorten time to recovery. A series of systematic Cochrane reviews have examined the evidence on other surgical and physical treatments. The 2011 Cochrane review of tailored facial exercises concluded that there is some evidence they can help improve outcome and reduce sequelae; further randomised controlled trials were recommended. We are currently updating this 2011 review to identify further published trials of facial exercise therapy.

**The FRAME study (*Facial Remote Activity Monitoring Eyewear*)**

A major study has been funded by NIHR i4i programme to develop a digital technology which could improve access & outcomes for patients referred for facial exercise therapy: see [*LINK provided*].

**National Survey**

As part of the FRAME study, we are undertaking a national survey to gather evidence on current treatment pathways for facial palsy patients and the place of facial exercise therapy. Various groups are being surveyed to include patients and their relatives, facial therapy specialists and medical staff (GPs, surgeons, hospital physicians).

**Your contribution**

We would be very grateful if you would help us by completing this online survey, answering all the questions applicable to you and giving as much detail as possible. A pilot indicates that this should take approximately 15 minutes to complete.

Please feel free to give any additional information or feedback at the end of the survey. All information you give us will remain anonymous and no information will be used which could identify you personally. If you have any questions or would like to talk about the survey, please contact [*LINK provided*]. We would also be happy to hear any additional thoughts or comments you may have.

Thank you very much for your help. Your support is very much appreciated and the more of you who reply, the more your opinions and experiences can be incorporated into our research.

**Q 1: Your connection to facial palsy (please tick one only):**

I am a parent/carer of a child under 18 with facial palsy

I was born with facial palsy

I acquired facial palsy during childhood

I am an adult with acquired facial palsy

I am a health professional with an interest in facial palsy

Another personal connection to facial palsy (e.g. partner, sibling, friend)

**Q 2: Your experience of facial palsy:**

## Q2.1 How many years’ experience of facial palsy do you have?

 Enter number

## Q2.2 At what stage were you (or your relative/friend) first reviewed by a health professional?

 Approximate time after first occurrence (please specify)

## Q2.3 What was the cause of the facial palsy (if known)?

Don’t know

Acoustic neuroma/vestibular schwannoma

Bell’s palsy

Birth trauma

Congenital facial palsy

Facial nerve neuroma

Lyme disease

Moebius syndrome

Neurofibromatosis type 2 (NF2)

Ramsay Hunt syndrome

Salivary gland/parotid tumour

Stroke

Other (please specify)

## Q2.4 At what stage did you (or your relative) first receive treatment (please tick only one)?

Within 72 hours following first occurrence of symptoms

Within 1 month of onset

1-6 months post onset

6-9 months post onset

More than 9 months post onset

Other (please specify)

## Q2.5 Which of the following treatments have been provided to date (please tick as many as apply)?

Prednisolone or other corticosteroids

Antivirals

Antibiotics

Advice on eye care

Botox injections

Plastic surgery e.g. face lift, brow lift, eyelid surgery, facial sling

Facial exercise therapy

Electrical stimulation therapy

Psychological therapy e.g. CBT

Other (please specify)

## Q2.6 Has a multidisciplinary clinic for facial palsy patients ever discussed this case?

Yes

No

Don’t know

**Q 3: Your experience of facial exercise therapy:**

## Q3.1 Have you (or your relative) ever been referred for facial exercise therapy?

Yes

No (If No, please **go to Q4**)

## Q3.2 Who referred you/ your relative for facial exercise therapy?

My General practitioner (GP)

Surgeon

Neurologist

ENT Specialist

Other (please specify)

## Q3.3 At what point did a referral for facial exercise therapy take place?

Within 1 month following first occurrence of symptoms

1-6 months post onset

6-9 months post onset

More than 9 months post onset

Other (please specify)

## Q3.3 To which centre was the referral for facial exercise therapy?

Birmingham

Chelmsford Essex

Glasgow Royal Infirmary

Liverpool

London Guys Hospital

London Queens Square

Manchester Lindens Clinic (NHS funded)

Newcastle

Norwich

Oxford

QVH East Grinstead

Southampton

Other (please specify)

## Q3.3 How long was the wait for an appointment, once referred?

 Enter number of weeks

## Q3.4 Were there any problems being referred for facial exercise therapy?

No

Yes (If Yes, please expand below)

| **What type of problem/ difficulty – please describe briefly** |
| --- |

## Q3.5 Was feedback provided during facial exercise therapy on level of improvement?

No

Yes (Please expand below)

| **Please describe briefly what information was provided, when & how** |
| --- |

## Q3.6 How would you describe your (or your relative’s) adherence to the facial exercise programme?

Very high

Medium to high

Poor to medium

Don’t know

| **Please briefly describe reasons for this response** |
| --- |

**Q3.7 Was facial therapy offered in conjunction with any other treatment (please tick as many as apply)?**

Botox injections

Electrical stimulation therapy

Other (please specify)

**Q 4: In your view, how important is it for the NHS to provide the following treatments for people with facial palsy [on a scale from 1 (very low value) to 10 (very high value)]:**

(***Please enter your rating for treatments where you have some experience – you can add a comment if you wish. if you have no experience please enter NA***)

| Treatment | Scale (1-10) or NA | Comment |
| --- | --- | --- |
| Corticosteroids |  |  |
| Antivirals |  |  |
| Antibiotics |  |  |
| Advice on eye care |  |  |
| Botox injections |  |  |
| Plastic surgery^1^ |  |  |
| Facial exercise therapy |  |  |
| Psychological therapy^2^ |  |  |
| Other (please specify) |  |  |

^1^ Includes face lift, brow lift, eyelid surgery, facial sling

^2^ Includes cognitive behavioural therapy (CBT)

**Q 5: Where do you live in the UK?**

London

South East England

East of England

South West England

West Midlands

East Midlands

Yorkshire and the Humber

North West England

North East England

Scotland

Wales

Northern Ireland

Other (please specify)

**Q 6: Thank you very much for your valuable contribution to this study.**

## Q6.1 We may wish to contact you for further details following this questionnaire. Please let us know whether you are willing to participate in a telephone interview? (all responses would be confidential and anonymous)?

No

Yes (If Yes, please provide details below)

Please provide your contact details - e-mail address (this will not be retained after the study is complete)

|  |
| --- |

| **If you have any additional comments, we would be very pleased to hear them.** |
| --- |

**Thank you for taking the time to complete our survey.**

# Multimedia Appendix 1 (contd)

# **2. Clinician Questionnaire**

**Facial Palsy – Introduction of Digital Technology**

Facial palsy affects approximately 22,500 people annually in the UK, with the cumulative number of cases since 2000 totalling over one third of a million. Although seventy per cent of cases will achieve complete recovery, it is estimated that the number of people living life with some level of disability during this period is 115,000, including 63,500 with a permanent deficit of facial function

Facial palsy affects both men and women equally, occurring most commonly in people aged between 15 and 60 years, but the condition is commoner in those who are pregnant, have diabetes, or conditions such as hypertension, sarcoidosis and HIV infection. It is estimated that for the 63,500 with a permanent defect, poor recovery leads to the loss of two quality adjusted life years (QALYs) per patient.

**Current treatment options for facial palsy**

Currently, oral corticosteroids (Prednisolone) within 72 hours of onset of symptoms are the only treatment recommended by NICE, with strong evidence that this can improve outcome and shorten time to recovery. A series of systematic Cochrane reviews have examined the evidence on other surgical and physical treatments. The 2011 Cochrane review of tailored facial exercises concluded that there is some evidence they can help improve outcome and reduce sequelae; further randomised controlled trials were recommended. We are currently updating this 2011 review to identify further published trials of facial exercise therapy.

**The FRAME study (*Facial Remote Activity Monitoring Eyewear*)**

A major study has been funded by NIHR i4i programme to develop a digital technology which could improve access & outcomes for patients referred for facial exercise therapy: see [*LINK provided*].

**National Survey**

As part of the FRAME study, we are undertaking a national survey to gather evidence on current treatment pathways for facial palsy patients and the place of facial exercise therapy. Several groups are being surveyed to include facial therapy specialists, medical staff (GPs, surgeons, hospital physicians) and patients.

**Your contribution**

We would be very grateful if you would help us by completing this online survey, answering all the questions applicable to you and giving as much detail as possible. A pilot indicates that this should take approximately 15 minutes to complete.

Please feel free to give any additional information or feedback at the end of the survey. All information you give us will remain anonymous and no information will be used which could identify you personally. If you have any questions or would like to talk about the survey, please contact [*LINK provided*]. We would also be happy to hear any additional thoughts or comments you may have.

Thank you very much for your help. Your support is very much appreciated and the more of you who reply, the more your opinions and experiences can be incorporated into our research.

**Q 1: Your clinical background (please tick one only):**

I am a Facial Therapy Specialist with the following training:

Physiotherapist

Speech & Language Therapist (SLT)

Occupational Therapist

Other (please specify)

Other involvement in facial palsy (please specify)

**Q 2: Your experience of treating facial palsy:**

## Q2.1 How many years’ experience of treating facial palsy do you have?

Enter number

## Q2.2 In an average year, APPROXIMATELY how many new patients are assessed by your team for facial therapy?

Enter number

## Q2.3 When you first see a new patient, have they received any of the following treatments prior to referral to you (please tick as many as apply)?

Prednisolone or other corticosteroids

Advice on eye care

Botox injections

Plastic surgery e.g. face lift, brow lift, eyelid surgery, facial sling

Facial exercise therapy

Psychological therapy e.g. CBT

Other (please specify)

## Q2.4 Which of the following do you regularly provide for patients (please tick as many as apply)?

Advice/ education about facial palsy

Massage, stretching, trigger point release, relaxation

EMG biofeedback, taping

Eye care, mouth care

Neuromuscular retraining

Botox clinics

Synkinesis delinking exercises

Other (please specify)

## Q2.5 Which of the following do you refer your patients for (please tick as many as apply)?

Opthalmology

Botox injections

Surgery for dynamic facial reanimation

Psychological therapy e.g. CBT

Other (please specify)

## Q2.6 Do you ever participate in a multidisciplinary clinic for facial palsy patients?

Yes

No

**Q 3: Your experience of facial exercise therapy referrals:**

## Q3.1 From which of the following areas do you receive referrals for facial exercise therapy (please tick as many as apply)?

South East – London

South East England

East of England

South West England

West Midlands

East Midlands

Yorkshire and the Humber

North West England

North East England

Scotland

Wales

Northern Ireland

Other (please specify)

## Q3.3 On average, how long do patients wait for their first appointment, once referred to you for assessment?

Enter number of weeks

## Q3.4 Have patients encountered problems in being referred for facial exercise therapy

No

Yes (If Yes, please expand below)

| **What type of problem/ difficulty – please describe briefly** |
| --- |

## Q3.5 At what stage are patients usually referred for facial exercise therapy (please tick as many as apply)?

Within 1 month following first occurrence of symptoms

1-6 months post onset

6-9 months post onset

More than 9 months post onset

Other (please specify)

## Q3.6 What percentage of your patients are referred by the following (only a rough estimate is required)?

General practitioner (GP)

ENT Specialist

Neurologist

Surgeon

Other (please specify)

## 3.7 Do you routinely provide feedback to GPs or other medical staff monitoring progress and outcomes for patients during facial exercise therapy?

No

Yes Final Outcome only (Please expand below)

Yes Progress & Final Outcome (Please expand below)

| **Please describe briefly how information is accessed/ provided** |
| --- |

## Q3.8 How would you describe patients’ level of compliance with their facial exercise programme?

Very high

Medium to high

Poor to medium

Don’t know

| **Please briefly describe reasons for your answer, any changes over time (early/ongoing compliance), any methods used to encourage patients, other factors.** |
| --- |

**Q 4: In your view, how important is it to provide the following treatments for patients with facial palsy [on a scale from 1 (very low value) to 10 (very high value)]:**

(***Please enter your rating for treatments where you have some experience – you can add a comment if you wish. if you have no experience please enter NA***)

| Treatment | Scale (1-10) | Comment |
| --- | --- | --- |
| Corticosteroids |  |  |
| Advice on eye care |  |  |
| Botox injections |  |  |
| Plastic surgery^1^ |  |  |
| Facial exercise therapy |  |  |
| Psychological therapy^2^ |  |  |
| Other (please specify) |  |  |
| Other (please specify) |  |  |
| Other (please specify) |  |  |

^1^ Includes face lift, brow lift, eyelid surgery, facial sling

^2^ Includes cognitive behavioural therapy (CBT)

**Q 5: What is your work location in the UK:**

Birmingham

Liverpool

London Guys Hospital

London Queens Square

Norwich

Oxford

QVH East Grinstead

Southampton

Other (please specify)

**Q 6: Thank you very much for your valuable contribution to this study.**

## Q6.1 We may wish to contact you for further details following this questionnaire. Please let us know whether you are willing to participate in a telephone interview? (all responses would be confidential and anonymous)?

No

Yes (If Yes, please provide details below)

Please provide your contact details - e-mail address (this will not be retained after the study is complete)

|  |
| --- |

| **If you have any additional comments, we would be very pleased to hear them.** |
| --- |

**Thank you for taking the time to complete our survey.**
